# Cannabinoids: Therapeutic Use in Clinical Practice

**DOI:** 10.3390/ijms23063344

**Published:** 2022-03-19

**Authors:** Cristina Pagano, Giovanna Navarra, Laura Coppola, Giorgio Avilia, Maurizio Bifulco, Chiara Laezza

**Affiliations:** 1 Department of Molecular Medicine and Medical Biotechnology, University of Naples Federico II, Via Pansini 5, 80131 Naples, Italy or cristina.pagano@unina.it (C.P.); vanna.navarra@libero.it (G.N.); coppola.laura6@gmail.com (L.C.); giorgioavilia@outlook.it (G.A.); 2 Institute of Endocrinology and Experimental Oncology, IEOS CNR, Via Pansini 5, 80131 Naples, Italy

**Keywords:** cannabinoids, cancer, neurodegenerative diseases, skin disorders, viral infections

## Abstract

Medical case reports suggest that cannabinoids extracted from *Cannabis sativa* have therapeutic effects; however, the therapeutic employment is limited due to the psychotropic effect of its major component, Δ9-tetrahydrocannabinol (THC). The new scientific discoveries related to the endocannabinoid system, including new receptors, ligands, and mediators, allowed the development of new therapeutic targets for the treatment of several pathological disorders minimizing the undesirable psychotropic effects of some constituents of this plant. Today, FDA-approved drugs, such as nabiximols (a mixture of THC and non-psychoactive cannabidiol (CBD)), are employed in alleviating pain and spasticity in multiple sclerosis. Dronabinol and nabilone are used for the treatment of chemotherapy-induced nausea and vomiting in cancer patients. Dronabinol was approved for the treatment of anorexia in patients with AIDS (acquired immune deficiency syndrome). In this review, we highlighted the potential therapeutic efficacy of natural and synthetic cannabinoids and their clinical relevance in cancer, neurodegenerative and dermatological diseases, and viral infections.

## 1. Introduction

**Figure 1:**
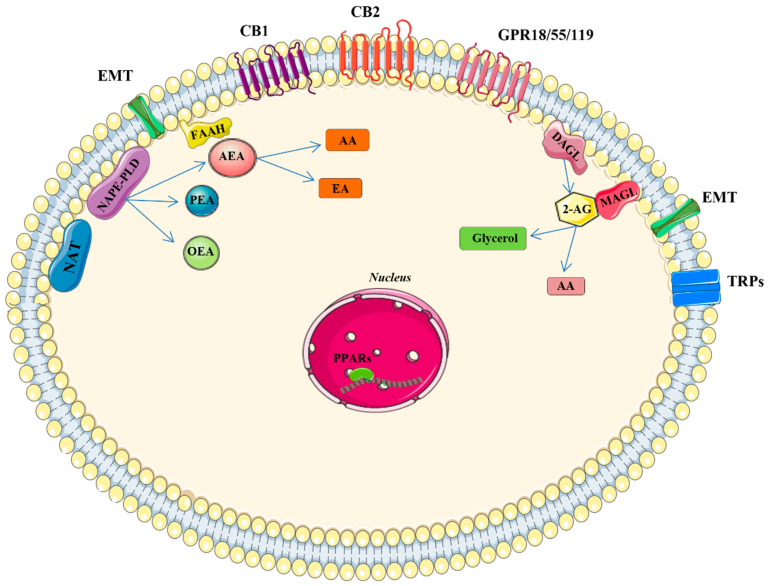
**Main components of the endocannabinoid system (ECS).** Biosynthesis of AEA and 2-AG take place on demand from membrane phospholipids by NAPE-PLD or calcium-dependent NAT and DAGL, respectively. The reuptake of endocannabinoids into cells occurs across the cell membrane by putative endocannabinoid protein transporters (EMT). FAAH is the main enzyme responsible for AEA degradation, while MAGL is the key enzyme in the hydrolysis of the 2-AG releasing arachidonic acid. Receptor targets of AEA and 2-AG on the plasma membrane are various: CB1, CB2, GPR18, GPR55, GPR119, and TRPs and in the nucleus PPARs. **Abbreviations**: EA: ethanolamine; AA: arachidonic acid; CB1: cannabinoid receptor 1, CB2: cannabinoid receptor 2, TRPs: transient receptor potential channels (V1-3, A1, and M8 types). GPRs: G-protein-coupled receptor (18, 55, and 119), and PPARs: peroxisome proliferator-activated nuclear receptors α, γ, or δ; PEA: palmitoylethanolamide; OEA: oleoylethanolamide.

**Figure 2:**
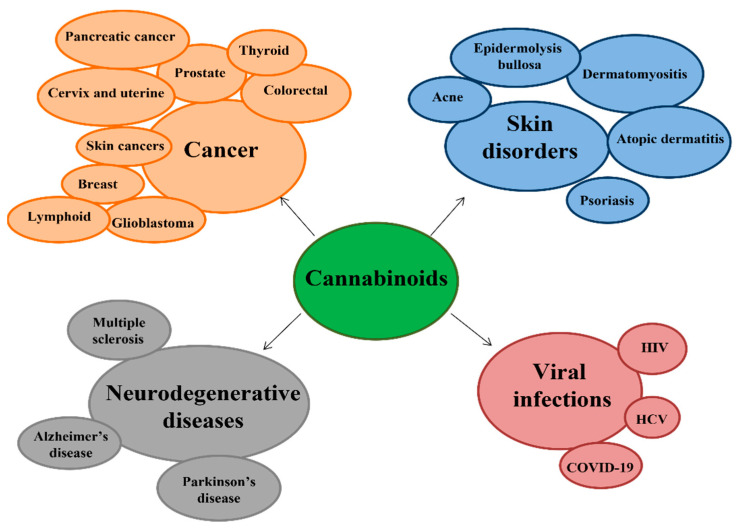
Diseases in which cannabinoids exhibit their therapeutic effects.

Cannabis contains more than 500 compounds, of which at least 100 are known to be cannabinoids called phytocannabinoids. The most abundant are Δ-9-tetrahydrocannabinol (THC), with psychoactive activity, cannabidiol (CBD) which is non-psychoactive, flavonoids, and terpenes. Phytocannabinoids, deriving from trichomes present in the female plants of *Cannabis sativa* [1], have led to the identification of the cannabinoid receptors CB1, mainly expressed in the central nervous system (CNS), and CB2, predominantly expressed in immune cells of the endogenous ligands anandamide (AEA) and 2-arachidonylglycerol (2-AG), and later, of the enzymes responsible for the synthesis of N-acyltransferase (NAT), N-acyl-phosphatidylethanolamine-hydrolyzing phospholipase (NAPE-PLD), and fatty acid amide hydrolase (FAAH) for the degradation of AEA; and diacylglycerol lipase (DAGLa/b) for the synthesis and monoacylglycerol lipase (MAGL) for degradation of 2-AG, thus constituting the endocannabinoid system (ECS) [2,3,4,5] (Figure 1). The endocannabinoid system is a complex molecular/biological system distributed throughout the body, playing critical roles in multiple physiological processes for maintaining an internal balance in the brain, skin, digestive tract, and liver, and the respiratory, cardiovascular, and reproductive systems, by regulating brain development, neurotransmitters, and cytokine release from microglia and directly influencing emotional behavior, cognition, fertility, and pregnancy. Moreover, it has been discovered that the alterations in the components of ECS are involved in several pathological diseases such as cancer and neurodegenerative and cardiovascular diseases; for this reason, the pharmacological modulation of the system has interested the research in medicine, allowing the drug discovery and development capable of affecting the signaling pathways downstream of the endocannabinoid system [6]. In this review, we will gather the clinical data about the therapeutic use of cannabinoids in cancers, neurodegenerative and dermatological diseases, and viral infections (Figure 2).

## 2. Phytocannabinoids: An Overview

The two major pharmacologically relevant compounds of Cannabis are THC and CBD. THC, the major psychotropic component, is able to activate the CB1 receptor by inhibiting the intracellular synthesis of cyclic adenosine monophosphate (cAMP) through a G-protein-mediated mechanism [2]. The activation of the CB1 receptor by THC is commonly associated with the psychoactive effects of Cannabis: hypolocomotion, hypothermia, catalepsy, and analgesia. On the other hand, THC has neuroprotective, antispasmodic, and anti-inflammatory actions, which are mediated through the activation of different receptors, such as CB2 and PPARγ [1]. It is a partial agonist of CB1 and CB2 receptors [7], showing a mixed agonist–antagonist profile depending on the cell type, expression of receptors, and presence of endocannabinoids or other full agonists [8]. THC, via CB1 receptor activation, inhibits both PI3K/Akt and RAS-MAPK/ERK survival pathways in colorectal carcinoma cell lines. It has a robust anti-proliferative potential in breast cancer cells as well as lung metastases and can inhibit the cell growth in vitro and in vivo of many tumors, such as myeloma, leukemia, melanoma, and hepatocellular carcinoma [9,10]. THC acts as a potent agonist for the putative cannabinoid receptors GPR18 and GPR55, without influencing ERK1/2 phosphorylation or β-arrestin recruitment. It is also an agonist of the vanilloid receptors TRPV2, TRPV3, and TRPV4 channels, while it does not affect TRPV1. Recently, it has been described to act as an agonist for the peroxisome proliferator-activated receptors (PPARs), specifically of the PPAR-γ subtype, a transcriptional factor of genes involved in energy homeostasis, lipid uptake, and metabolism [11]. Moreover, THC is highly lipophilic and accumulates in adipose tissue and the spleen which can act as long-term storage sites in fatty tissue and organs such as the heart, liver, and spleen [12]. It is metabolized in the liver where it is converted to 11-hydroxy-THC, which is reported to have psychoactive activity, or 11-nor-9-carboxy-THC [13]. THC taken orally usually peaks in the circulation within 1–2 h, with blood plasma levels lower than those obtained during smoking; it readily crosses the blood–brain barrier and can be found in high quantities in the brain [14]. In contrast, the non-psychoactive cannabidiol CBD does not affect motor and cognitive functions or body temperature. It displays a low level of activity for CB1 and CB2 receptors and it works as an inverse agonist for the human CB2 receptor contributing to its anti-inflammatory effects. CBD acts as an entourage molecule, reducing the psychotic effects of THC, such as tachycardia, anxiety, and hunger in humans and rats. In addition, several studies describe CBD as an interesting curative drug for cancer, diabetes, neurodegenerative disorders, and inflammation. Moreover, it inhibits the cellular uptake of the endogenous CB1 receptor ligand, AEA, directly affecting endocannabinoid tone. Furthermore, CBD shows cytotoxicity in breast tumor cells and is cyto-preservative for normal cells [15]. CBD has many cannabinoid-receptor-independent properties. It is known that CBD has anti-inflammatory and immunosuppressive effects because it increases adenosine signaling through the inhibition of adenosine uptake [16]. The serotonin receptors have been involved in the therapeutic effects of CBD. Several studies highlighted that it acts as a full (5-hydroxytryptamin) 5HT_1A_ agonist [17] inhibiting the uptake of serotonin, noradrenaline, dopamine, and GABA, which are thought to perhaps facilitate the anxiolytic properties of CBD. Lastly, CBD has physiologic properties that are not yet clearly related to a specific mechanism, such as antioxidant, anticonvulsant, analgesic, and immunomodulatory functions [16]. CBD is also an agonist of PPAR-γ, and of TRPV1 and TRPV2 [18]. CBD is highly lipophilic, has a poor oral bioavailability and accumulates in body fat. It is metabolized in the liver and the intestine by cytochrome (CYP) P450, CYP2C19, and CYP3A4, and 5′-diphosphoglucuronosyltransferase (UGT) UGT1A7, UGT1A, and UGT2B7 isoforms, mainly producing hydroxylated and carboxylated metabolites. The main human metabolite is 7-carboxy-cannabidiol (7-COOHCBD) and represents the ~90% of all drug-related substances measured in the plasma. The primary excretion route of CBD is through feces (84%), followed by urine (8%) [19,20].

## 3. Other Phytocannabinoids with Therapeutic Potential

Δ8-Tetrahydrocannabinol is an isobaric and more stable isomer of Δ9-tetrahydrocannabinol (Δ9-THC). It displays psychoactive effects and partial agonism on CB1 and CB2 receptors. Recent evidence reveals that Δ8-THC produces antinociceptive effects in pre-clinical models with similar potency via the activation of CB1R [21]. Cannabinol is an oxidation product of Δ9-THC and is a weak psychoactive substance with a higher affinity for CB2 than CB1 receptors. This compound is a potent agonist of the transient receptor potential cation channel, subfamily A, member 1 (TRPA1), and an antagonist of the transient receptor potential cation channel, subfamily M, member 8 (TPRM8). Recently, it has been observed that cannabinol (CBN) influences disease progression but not survival in the mouse model of amyotrophic lateral sclerosis (ALS) [22]. Cannabigerol (CBG), a non-psychoactive phytocannabinoid, has a low affinity for the cannabinoid CB1 and CB2 receptors, but inhibits AEA uptake, affecting the ECS. The non-cannabinoid activity of CBG involves its ability to potently activate the α2 adrenergic receptor and moderately block the serotonin 5HT_1A_ receptor; moreover, it interacts weakly with TRPV1 and TRPV2 channels [9]. Potential targets of CBG actions include transient cyclooxygenase (COX-1 and COX-2) enzymes. CBG was shown to exert antiproliferative, antibacterial, and anti-glaucoma actions and to antagonize the anti-nausea effect of CBD. Additionally, it exerts protective and curative effects in the model of murine colitis and has antioxidant effects [23]. Cannabichromene (CBC), the most abundant phytocannabinoid in the plant, displays a low affinity for CB1 and CB2 receptors, but affects the endocannabinoid system by inhibiting AEA uptake. The more relevant pharmacological activity of CBC explored so far is at TRP channels. Among the phytocannabinoids, it is the most potent agonist of the TRPA1 channels, and with a lower potency, it is also able to activate TRPV3 and TRPV4 and block TPRM8 receptors with the same cellular and functional outcome [9]. CBC has antinociceptive and anti-inflammatory effects in vitro and in vivo. CBC decreased carrageenan-induced and LPS-induced inflammation in rats and mice, and modestly inhibited thermal nociception and potentiated THC antinociception in mice [24]. Δ9-Tetrahydrocannabivarin (Δ 9-THCV) is an an-propyl analogue of Δ9-THC. It has been shown that this constituent of Cannabis can act in both in vitro and in vivo experiments as a CB1 receptor antagonist and as a CB2 receptor partial agonist, while, at higher doses, the in vivo effects indicate CB1 agonism in an antinociception model. In addition, THCV has been reported to target GPR55 receptors. Beyond the endocannabinoid system, it has been reported to activate 5HT_1A_ receptors, as well as different TRP channel subtypes [25]. Cannabidivarin (CBDV) is an an-propyl analogue of cannabidiol that lacks psychoactive properties. This compound displays a very weak affinity for CB1 receptors, but it has been observed to have a certain affinity and activity for CB2 receptors, depending on the different experimental conditions used. It acts as an inverse agonist of GPR6 and as an antagonist of GPR55. CBDV is also an agonist of TRPA1, TRPV1, and TRPV2 channels, and an antagonist of rat TRPM8 channels. Recently, it has been shown that CBDV can act as a functional partial agonist for dopamine D2-like receptors in vivo, suggesting for the first time a possible role of dopamine signaling in the effect of this phytocannabinoid. Moreover, this substance exerted anticonvulsant effects in three out of four in vivo rodent models of seizures and human studies and, recently, in few preclinical models of autism spectrum disorders (ASD). In addition, CBDV attenuates intestinal inflammation in a model of inflammatory bowel disease, and it reduces the consequences of inflammation in muscles of dystrophic mice, likely interacting with TRP channels [26].

## 4. FDA-Approved Cannabinoids in Humans

The Food and Drug Administration (FDA) has approved one Cannabis-derived drug, Epidiolex (cannabidiol), and three synthetic Cannabis-related drugs, Cesamet (nabilone), Marinol (dronabinol), and Syndros (dronabinol). Nabilone (Cesamet; Valeant Pharmaceuticals North America) and dronabinol (Marinol; Solvay Pharmaceuticals) are synthetic analogues of Δ9-THC [27]. Clinical studies demonstrated the efficacy, safety, and tolerability of nabilone in reducing nausea and vomiting in cancer patients, demonstrating its efficacy as a rescue or adjunct therapy for cancer patients. The current pharmaceutical form of nabilone consists of capsules in strengths of 0.25, 0.5, and 1 mg. Recently, the effectiveness of nabilone has been addressed in the treatment of neuropathic and chronic pain, and of spasticity related to MS [28]. Dronabinol is evaluated for its analgesic properties in patients with bone metastases from breast cancer (early phase I study; NCT03661892), and as an adjunct therapy to opiates in patients with chronic pain (NCT00153192). Dronabinol is administered as an appetite stimulant in AIDS wasting syndrome and for combating cancer chemotherapy-induced nausea and vomiting. Recent and ongoing clinical trials suggest that dronabinol and nabilone were explored for the treatment of Cannabis Use Disorder (CUD) with positive effects. Another area of interest is the treatment of post-traumatic stress disorder (PTSD) in military personnel with THC or nabilone [29]. Epidiolex contains >98% CBD and less than 0.15% THC; it is a solution orally administered for the treatment of two forms of severe childhood epilepsy (Dravet syndrome and Lennox–Gastaut syndrome). Lennox–Gastaut syndrome (LGS) [30] is a drug-resistant form of epilepsy that begins in early childhood and is characterized by cognitive impairment. Clinical studies have described that Epidiolex significantly reduces the seizure frequency in treated patients [31]. Similarly, Dravet syndrome (DS) is a developmental disorder characterized by severe seizures and delayed onset of psychomotor deficits [32] and affects motor and cognitive development. Epidiolex reduces convulsive seizure frequency in patients after 4 weeks of treatment with 20 mg/kg/day [31]. Sativex^®^ (United States Adopted Name (USAN), nabiximols) (GW Pharmaceuticals, Sovereign House Vision Park, Chivers Way, Histon, Cambridge, UK) is a Cannabis-based pharmaceutical product containing THC and CBD in approximately a 1:1 ratio, delivered in an oromucosal spray. It is approved in the UK, Germany, and Switzerland for multiple sclerosis (MS)-related spasticity and in Canada for pain associated with MS and cancer [33].

## 5. Therapeutic Applications of Cannabinoids

### 5.1. Cancer

Cancer is a disease characterized by the rapid proliferation of abnormal cells that grow beyond their usual boundaries. Tumor transformation is a multi-stage process that starts mainly after DNA damage, leading to mutations, cell cycle defects, and the inhibition of apoptosis [34]. Considering the wide distribution of the cannabinoid system, the role of these substances in cancer has been the focus of the last years of research. In the oncology field, the applied clinical use of cannabinoids is primarily as palliation of therapy- and tumor-related symptoms, and their study in this sense had already started during the early 1970s [35]. It is known that the side effects of chemotherapy (i.e., pain, nausea, vomiting, muscle spasm, insomnia, appetite changes, and waste) that accompany this disease are multiple and, in a lot of cases, devastating, due to the mechanism of action of the drugs used and the characteristics of the cancer itself. As said before, the ECS is found in different areas of the nervous system, such as the basal ganglia, the cerebellum, and the spinal cord, explaining their effects on memory, emotion, movement, and pain transmission [36]. Cancer patients are often affected by chronic pain and rely heavily on opioid analgesics that, depending on the state or the genetics of the individual, can lead to a plethora of serious problems, the most important ones being drug dependence and improper dosage [37,38]. One of the earliest medical applications of cannabinoids is the regulation of pain [39]. Recent studies highlighted that the ECS is indeed active on all levels of nociceptive transmission, targeting preferentially affective components of pain, due to the frontal and limbic distribution of CB receptors in the brain [40,41]. CB1, specifically, is defined as the receptor with the main role in the analgesic effects of cannabinoids, although CB2 also appears to be marginally involved [42]. Due to this, Cannabis derivatives began to be used as adjuvants or, in some cases, as substitutes for opioid treatment [38]. In Europe, dronabinol, also known as delta-9-tetrahydrocannabinol (2.5 mg and 5 mg gel capsules containing THC in sesame oil), is used as an analgesic in cancer-related chronic pain, while its efficacy in acute pain is less conclusive [43]. Several studies are evaluating nabiximols (marketed as Sativex^®^) for the management of cancer-related pain, but the results are variable and not always decisive. Despite this, Sativex has been approved in Canada as an analgesic treatment in adult patients with advanced cancer and persistent pain who no longer respond to the highest tolerated dose of strong opioids [44,45]. One of the most distressing acute side effects of chemotherapy is emesis, that can severely impact the patient’s quality of life and can result in anorexia, metabolic derangements, and the failure of antineoplastic treatment [46]. Cannabinoids are well-known anti-emetic drugs. Physiologically, CB1 receptors, again the main proponents of the effect in this process, attenuate the emetic reflex by inhibiting the release of excitatory transmitters [47]. Notably, CB1R can be found on dopaminergic, noradrenergic, and other neurons situated in the brain regions regulating nausea and vomiting, which even now are still not completely described [48,49]. Nabilone (as 0.25, 0.5, and 1 mg tablets), is administered for chemotherapy-induced emesis in patients who have failed to respond to conventional antiemetic treatments [50]. Similarly, dronabinol is FDA-approved for the treatment of chemotherapy-induced emesis, with a recommended starting dose of 5 mg every 1–3 h prior to chemotherapy followed by 5 mg every 2–4 h [44,51]. It is important to highlight that the use of cannabinoids, especially of the THC component, has to be closely supervised and controlled due to the potential side effects that can be experienced by the patients, such as psychotomimetic reactions, an alteration of the mental state (dysphoria, euphoria, anxiety, or panic reactions in some new users), and some limited toxicity, associated mainly with synthetic cannabinoids [52]. In the last few years, there have been numerous studies that have investigated natural and synthetic (endo)cannabinoids for targeting and killing tumors, to the point of collecting overwhelming evidence to suggest that cannabinoids can be used as adjuvant agents for the treatment of cancer. Brain, prostate, colorectal, breast, uterine, cervix, thyroid, skin, pancreatic, and lymphoid cancers are some of the various tumor types that have been studied to be more or less severely affected by the action of cannabinoids. Pisanti et al. and Ladin et al. have collected very efficiently the various scientific works in which the in vitro and in vivo action of cannabinoids in cancer has been ascertained [53,54]. The reason behind this strong interest lies in the vast number of cancer-related pathways that are modulated by cannabinoid agonists once they bind to the canonical CB1 or CB2 receptors, a process that can lead to the block of the cell cycle, the inhibition of cell proliferation, and lastly to cell death. As said before, cannabinoids also act through other receptors, such as theTRPV1, or can be completely receptor-independent [55]. Hence, some of the Cannabinoid-related drugs’ effects are suggested to be the result of the inhibition of the PI3K-Akt pathway and the activation of the MAPK pathways, resulting in apoptotic death. They can also induce the de novo synthesis of ceramide, a pro-apoptotic sphingolipid, that in turn activates an ER stress-related signaling pathway, which leads to the inhibition of the AKT/mTORC1 axis, and, thus, death by autophagy [56]. Furthermore, cannabinoids exert anti-angiogenesis effects, mainly by blocking the activation of the vascular endothelial growth factor (VEGF) pathway, an inducer of angiogenesis [57], as much as by affecting several factors involved in this process [58]. Anti-invasiveness and anti-metastasis actions have also been demonstrated to be the result of THC and CBD treatment in several studies [59,60]. Based on the large body of scientific literature that has been accumulated over the years, where cannabinoids’ anti-cancer potential has already been well established, a wider number of clinical studies should be encouraged. Some trials have already started, especially on glioblastoma multiforme, where cannabinoids have been seen to be particularly efficient. A recent study conducted by Twelves et al. concluded that nabiximol spray combined with Temozolomide appeared tolerable to the glioblastoma patients selected, with a difference in survival rate (83% after 1 year of nabiximol treatment) when confronted with placebo-treated patients (44% after 1 year) [61]. Even before that, a first pilot trial tried to investigate the effects of intracranial THC administration in patients with recurrent glioblastoma, showing a reduction in tumor proliferation in two of the nine patients recruited [62]. These studies, although limited, still support the idea of further developing and expanding clinical studies to test them as single drugs or, ideally, in combination with other therapies.

### 5.2. Neurodegenerative Diseases

Neurodegeneration is the main cause of progressive deterioration of cognition and memory of Parkinson’s, Alzheimer’s disease, and multiple sclerosis.

Alzheimer’s disease (AD) is caused by extracellular deposits of β-amyloid plaques and neurofibrillary tangles composed of hyperphosphorylated tau protein, and reduced levels of choline acetyltransferase [63]. Additional pathologies include functional mitochondrial defects, increased oxidative stress (OS), neuroinflammation, and the failure of enzymes involved in energy production, causing nerve cell exhaustion. It has been described that an activation of microglia in plaque-filled regions and cell death occurs due to excitotoxicity [64]. No therapeutic treatment is able to arrest the progressive dementia and cognitive decline [65]. The expression of ECS components is altered in animal models and human post-mortem samples of the AD brain, especially in the hippocampus and cerebral cortex. It has been extensively reported that there is a reduced level of the neuronal CB1 receptors [66], while a protective role of CB2 receptors against neuroinflammation has been suggested due to their upregulation in microglial cells in animal models. Moreover, CB2 activation attenuated the inflammation due to the release of neurotoxic and pro-inflammatory mediators by reactive astrocytes and microglial cells, thus modulating Aβ aberrant processing and stimulating microglial proliferation and migration. Additionally, in post-mortem studies, an increase in 2-AG levels was found in the hippocampus and in plasma of patients with AD [67]. Moreover, FAAH protein levels and activity are selectively overexpressed in glial cells, a characteristic that leads to the pro-inflammatory effects that accompany Alzheimer’s disease [68]. These studies suggest that modulation of the endocannabinoid system through the use of cannabinoids could protect AD individuals from excitotoxicity and neuroinflammation. Indeed, recent data have shown that CBD can counter Aβ-induced insults through reduction of oxidative stress, tau phosphorylation, and expression of inducible nitric oxide synthase [69]. Finally, experiments in mouse models have shown that when CBD and THC are used in combination, their efficacy is higher than the cannabinoids used alone [70]. Various clinical tests using cannabinoids were carried out to treat some consequences and co-morbidities of AD, such as anxiety, agitation, and depression. Nabilone reduced the severity of agitation in a 72-year-old man with cognitive decline [71]. A randomized trial of adjunctive dronabinol for the treatment of agitation in AD-afflicted patients has described that dronabinol was more effective than placebo in reducing agitation and was well-tolerated with adverse events not different than placebo [72]. Additionally, dronabinol was effective in 15 patients suffering from probable Alzheimer’s disease who were refusing food. The treatment reduced the severity of the altered behavior and this effect persisted during the placebo period in patients who first received dronabinol. Adverse reactions included euphoria, somnolence, and tiredness, but they did not require discontinuation of therapy [73].

Parkinson’s disease (PD) is a neurodegenerative disorder that leads to tremor and difficulty with walking and balance, while muscle rigidity can cause difficulties in starting and ending movements, symptoms which get worse over time. Other relevant symptoms may affect the psychological and behavioral aspect of this disease such as insomnia, depression, memory problems, and fatigue. The main pathological cause of PD is cell death in the basal ganglia, especially of dopaminergic neurons found predominantly in substantia nigra pars compacta (SNc), in neostriatum, and subthalamic nucleus [74]. It is characterized by the intracellular accumulation of Lewy bodies enriched in α-synuclein protein responsible for the motor symptoms in PD [75]. Other pathological mechanisms involved in the loss of dopaminergic neurons are oxidative stress, mitochondrial dysfunction, dysregulation of calcium homeostasis, and neuroinflammation [76]. It has been described that the expression levels of ECS elements are altered in this pathology. In an in vivo model based on parkinsonian monkeys treated with levodopa to induce dyskinesias, the expression of CB1 receptors was upregulated in the basal ganglia during the active phase of dyskinesia. Moreover, the globus pallidus of untreated parkinsonian monkeys showed a deep alteration of AEA-synthesizing/degrading enzymes [77]. Conversely, in post-mortem brain samples from patients with PD a reduced expression of CB1 receptors in some areas of the basal ganglia has been observed [78]. Analysis of cerebrospinal fluid samples from patients at different stages of PD have revealed increased levels of anandamide [79]. Higher levels of AEA have also been detected in animal models of PD and lower activity of the AEA membrane transporter and FAAH enzyme in several rat models [80]. Synthetic cannabinoids or phytocannabinoids were tested in mouse models of PD in various preclinical studies that revealed a neuroprotective effect of MAGL inhibitors such as JZL184, and blocking of CB1 receptor activity by the CB1 antagonist SR141716A. In addition, antiparkinsonian activity has been described of Δ9-THCV in a 6-hydroxydopamine-induced nigra lesion mouse model, delaying disease progression [81]. Numerous clinical studies highlighted the potential benefits of cannabinoids on motor symptoms such as akinesia, tremor, or dyskinesia of PD patients. A study conducted on Czech PD patients who have taken Cannabis buds orally reported a general improvement of PD symptoms such as a reduction of resting tremor and muscle rigidity and a decrease in bradykinesia. 5% of patients showed a worsening of symptoms. An observational study in 22 Israeli patients showed a significant improvement in resting tremor, rigidity, and bradykinesia, and the non-motor aspects sleep and pain [82]. In an open-label study on PD patients treated with CBD over a period of 4 weeks, Zuardi et al. reported a significant reduction in psychotic symptoms, such as illusions and hallucinations, and minor symptoms, such as withdrawal and depression [83]. The reported positive effects of CBD suggest that this drug could complement standard therapy for PD. In a randomized placebo-controlled study, the efficacy and safety of nabilone in patients with PD afflicted by troublesome non-motor symptoms (NMS), which include autonomic nervous system dysfunction, olfactory loss, disorders of mood and cognition, and sleep problems, were tested. The investigation highlighted the effectiveness of the treatment due to its ability to ameliorate anxiety and sleep problems [84]. In another pilot study, ultramicronized palmitoylethanolamide (um-PEA), administered to PD patients receiving levodopa therapy, produced beneficial effects in most non-motor and motor symptoms. um-PEA slowed down disease progression and disability in PD patients [85].

Multiple Sclerosis is a progressive autoimmune-mediated neurodegenerative process of the central nervous system characterized by lesions in focal areas of demyelination of the axon and inflammation in the white matter. The progressive neurodegeneration leads to neuronal dysfunction and to neurological symptoms dependent on the site of the lesions in the brain and spinal cord. The symptoms include spasticity, painful spasms, weakness, ataxia, optic neuritis, dysphagia balance problems, fatigue, and incontinence [86]. Several data have revealed altered expression of the ECS elements in MS. In human post-mortem samples of MS donors, the expression of CB1 and CB2 receptors was increased. Interestingly, in plasma samples from patients with MS with different phenotypes of the disease (relapsing remitting MS, primary progressive MS, secondary progressive MS (SPMS), and progressive relapsing MS), an increase in the expression of both CB1 and CB2 receptors was found in patients with primary progressive MS. However, the levels of several endocannabinoids (AEA, palmitoylethanolamide, and oleoylethanolamide) were increased in all MS phenotypes. The levels of AEA were high in secondary progressive MS, probably due to the reduction of FAAH expression. In relapsing remitting MS samples, an increase in N-arachidonoyl NAPE activity and a decrease in FAAH activity were detected, without any differences in 2-AG levels [87]. In some studies, blood levels of endocannabinoids of MS patients were increased and CSF levels were decreased, while in other studies, AEA levels were increased in the CSF as well as in peripheral lymphocytes and the brain [88]. Studies in mouse models of MS have also found elevated AEA in brain and spinal cord samples. Moreover, in mouse models it has been described that CB1R activation ameliorated tremor and spasticity, while CB1 and CB2 agonists improved clinical scores via immunomodulatory and anti-inflammatory mechanisms [89]. Finally, in murine models of MS, it has been observed that the inhibition of FAAH activity reduced spasticity. Such an effect could be due to an increase in AEA levels, suggesting a potential role of this endocannabinoid in MS pathology. Interestingly, MAGL inhibitors reduced neuronal excitotoxicity preventing demyelization [81]. In a recent study, the effect of CBD in an experimental autoimmune encephalomyelitis (EAE), a murine model of MS, has been elucidated. CBD attenuated neuroinflammation through a decrease in pro-inflammatory cytokines and induction of anti-inflammatory cytokines and the gain of myeloid-derived suppressor cells [90]. The therapeutic efficacy of Sativex has been evaluated in several clinical trials as an add-on therapy for the management of moderate and severe spasticity in patients with MS. Sativex is an oromucosal spray containing 27 mg of Δ9-THC and 25 mg of CBD/1.0 mL, in an aromatized water-ethanol solution [28]. It reduced spasticity improving the patient’s quality of life. A study showed that Sativex reduced neuropathic pains which is a common symptom affecting between 17% and 70% of patients of MS. Other clinical studies demonstrate that treatment with this substance did not cause a statistically significant decline of postural stability, cognitive performance, mood, or psychomimetic effects. In addition, Sativex has a low incidence of adverse reactions and was well-tolerated in patients with MS [12]. In a randomized study, the effect of ultramicronized palmitoylethanolamide (um-PEA) in patients with MS has been evaluated. It added to IFN-β1a in the treatment of relapsing remitting multiple sclerosis. Patients with MS perceived an improvement in pain sensation and of quality of life. The treatment modulated the levels of some pro-inflammatory cytokines, reducing the circulating levels of interferon-γ, the serum levels of tumor necrosis factor-α and interleukin-17, and finally the plasma levels of N-acylethanolamine when compared with the placebo group [91].

### 5.3. Skin Disorders

The ECS has garnered significant attention in recent years for its therapeutic potential for various pathologies, including in the skin, and there is an increasing interest in their beneficial effects in clinical applications [92]. The ECS maintains skin homeostasis by regulating many aspects of cell proliferation, differentiation, and inflammatory signalling [93]. CBD, the non-psychoactive compound from the Cannabis plant, may be efficacious for some skin disorders, such as eczema, psoriasis, pruritis, and inflammatory conditions, but the confirmation of the clinical efficacy and the elucidation of its underlying molecular mechanisms have yet to be fully identified [92]. The ECS binds CB1 and CB2 receptors present in epidermal keratinocytes, cutaneous nerve fibers, dermal cells, melanocytes, eccrine sweat glands, and hair follicles. The CB receptors remain the primary targets for the ECS but they also have the ability to bind TRPs located in various types of skin cells and are involved in different functions such as the formation and maintenance of the skin barrier, cell growth, cell differentiation, and immunological and inflammatory processes [94]. For this reason, it is plausible that treatment with topical cannabinoids could be efficacious for certain disorders or skin health in general. Most of the clinical evidence to date has focused on the effects of CBD and other cannabinoids when consumed, inhaled, or injected. There is limited research investigating the therapeutic potential for topical applications. Yet, there is evidence to suggest that applying cannabinoids, and specifically CBD, topically may be a viable route of administration for certain conditions [95]. Along these lines, it is important to note that according to a recent observational study reporting three cases of self-initiated topical CBD use in patients with epidermolysis bullosa (EB), CBD may improve quality of life in such patients. Indeed, one patient was weaned completely off oral opioid analgesics, and all three patients reported faster wound healing, less blistering, and amelioration of pain. The effects might have been due to the anti-inflammatory activity of CBD and might have beneficially modulated keratin expression [96,97]. Likewise, in another small pilot study, three EB patients, who were prescribed pharmaceutical-grade sublingually administered cannabinoid-based medicine (CBM) comprising THC and CBD, reported improved pain scores, reduced pruritus, and decreased overall analgesic drug intake [98]. The effects of cannabinoids are tested, and still are today, in acne, a skin disease triggered by various processes such as seborrhoea, hormonal imbalances, immune reactions, and infectious and environmental factors [99]. It was demonstrated that phytocannabinoids safely decrease sebum production, inhibit sebocyte proliferation, and reduce the expression of pro-inflammatory cytokines as demonstrated in multiple in vitro and in vivo studies, including a human trial where topical application for 12 weeks showed good results in decreasing erythema and skin sebum [100,101,102]. A phase 2 trial enrolling over 360 participants is evaluating the effects of a topical cannabinoid named BTX 1503 (a solution made up of 5% CBD as the active ingredient) on acne lesions. Recently, it has been reported that all doses of BTX 1503 were very safe—no serious adverse events were detected, while positive effects on acne lesion reduction were clearly observed; in particular, a strong and consistent impact on inflammatory lesions was seen across the entire study with an even greater non-inflammatory lesion reduction [103,104].

Ajulemic acid (AJA), a novel synthetic cannabinoid that triggers the release of endogenous eicosanoids and decreases TNF-α, as well as IFN-α and IFN-β production, has been proven safe and effective in improving the clinical status of patients with scleroderma; the mechanisms were determined to be related to the reduction of inflammation-related gene expression, ascertained on skin biopsies [105,106]. Moreover, an international phase 3 clinical trial of AJA in scleroderma was initiated in 2018. In dermatomyositis, an inflammatory myopathy featuring skin rash and erythema, AJA is successfully undergoing phase 2 clinical trials when administered orally as capsules [105,106]. Another randomized controlled trial has reported a reduction in type 1 and 2 IFN levels and T-helper cell inflammation in patients with dermatomyositis treated orally with AJA for 12 weeks, compared to those receiving placebo [107]. In addition, a phase 3 study for testing the efficacy and safety of AJA in the treatment of dermatomyositis was launched in 2019 [108]. To date, the effectiveness of topical use of cannabinoids is known for CBD, PEA, and THC. The treatment with CBD, PEA and AEA for atopic dermatitis on a large cohort of patients with asteatotic eczema experienced improvement in scaling, dryness, and itch [109,110]. Moreover, topical adelmidrol, an analogue of PEA, appeared effective for the treatment of pediatric atopic dermatitis [111]. Following a treatment course of four weeks, all participants experienced a significant improvement in pruritus and erythema. Moreover, all subjects experienced symptom improvement of at least 70% in the following domains: dryness, excoriation, lichenification, scaling, erythema, and pruritus. Maida and Corban showed that topical combined CBD–THC appears to be effective for pain relief in patients with pyoderma gangrenosum; in fact, three patients achieved symptomatic relief of pain (*p* < 0.05) with an overall pain reduction of 30% [112]. Two studies reported first data about a total of six psoriasis patients that were treated with topical CBD or THC: all patients had a good response to treatment with a resolution of psoriasis plaques [113,114]. Numerous studies are underway to test the efficacy of new cannabinoids and to evaluate their clinical efficacy in known and still unknown skin diseases. The data obtained to date give us hope in their use as future therapeutic agents.

### 5.4. Viral Infections

Acquired Immunodeficiency Syndrome (AIDS) is the end-stage disease of human immunodeficiency virus (HIV) infection [115]. It is known that despite the administration of antiretroviral therapy (ART), HIV-affected patients have an increased risk of non-opportunistic complication such as cardiovascular, pulmonary, renal, and hepatic events due to persistent immune activation [116,117]. The pathogenesis and survival of HIV are related to chronic inflammation and immune activation, driven by the microbial translocation of bacterial products through the intestinal mucosa [118,119]. When ART is initiated in the early stage of HIV, intestinal integrity is not fully restored [120]. HIV reservoirs are the reason HIV is still an incurable infection. Although HIV can also persist in myeloid cells, CD4 + T cells are the best characterized and most abundant reservoirs [121,122,123]. Reducing immune activation and inflammation levels may be a potential therapeutic target. Cannabis has anti-inflammatory and anti-fibrotic properties and could be a valid method to reduce immune activation and improve the immune profile [124]. Cannabinoids, present in the hemp plant *Cannabis sativa*, have been recognized for centuries for their analgesic, anticonvulsant, bronchodilator, sedative, hypnotic, and antispasmodic properties [125,126]. Their biological activity is given by cannabinoid receptors CB1 and CB2 through the activation of heterodimeric G proteins that function as signaling and regulatory proteins to operate or modulate the intracellular signaling pathways [127,128]. Although CB1 receptors are mainly expressed in the central nervous system, they are also present in the lung, liver, and kidney. The endocannabinoid system also plays a key role in the neural and molecular control mechanisms of the gastrointestinal tract. In fact, the endocannabinoid system plays a key role in the normal physiological functions of the gastrointestinal tract, including motility, gut–brain-mediated fat intake, hunger signaling, inflammation, and intestinal permeability [129].

As with AIDS, cannabinoids are used to counteract symptoms such as anorexia, cachexia, nausea/vomiting, neuropathic pain, and adverse effects of antiretroviral therapy [130]. Cannabis is known for its effect on stimulating appetite through the activation of the CB1 receptor, and for this purpose, it has been used as a treatment for AIDS wasting syndrome. CB1 receptors play a pivotal role in nonalcoholic fatty liver disease and alcoholic liver disease. CB2 receptors have been reported to have anti-inflammatory effects and could be useful in inflammatory liver disease. CB2 receptors express antifibrinogenic properties. Studies on CB2 agonist conducted on fibrotic rats produced several effects such as an improvement in liver fibrosis, a decrease in inflammation, and an increase in apoptosis of hepatic myofibroblasts [131,132,133,134,135]. Cannabinoids have been shown to inhibit productive HIV infection in primary human T lymphocytes, and a CB2 antagonist blocked this effect [136]. Interference with CXCR4 chemokine receptor signal transduction is considered to lead to a reduced accumulation of F-actin. This in turn prevents the movement of the viral pre-integration complexes towards the nucleus. It has also been hypothesized that CB2 agonists may inhibit anti-CD3/anti-CD28-induced T cell activation [136]. One of the drugs used for the treatment of HIV-associated wasting syndrome is dronabinol. Patients treated with this drug have experienced a significant improvement in appetite, although it is not accompanied by a significant improvement in weight, nausea, and mood versus placebo. Another synthetic cannabinoid with powerful anti-emetic properties is nabilone [137]. There are other medical Cannabis products used to treat HIV-associated sensory neuropathy (HIV-SN) [138,139,140,141].

Two randomized, placebo-controlled trials found that 28% of patients with HIV-SN achieved a clinically and statistically significant pain reduction (≥30% from baseline) with smoked Cannabis products, with a necessary number for treatment (NNT) of 4 [138,139,140]. Studies show that even smoked or ingested Cannabis, containing the THC component, is capable of improving appetite, weight, and mood, thus improving quality of life [142]. Despite this, recent studies have shown that chronic Cannabis smoking weakens the immune system leading to increased symptoms of chronic bronchitis, cough, sputum production, and wheezing [143,144,145]. Lung function, lung reactivity, and bronchial cell characteristics of Cannabis-only smokers have shown to be negatively affected by continuous usage [146,147]. HCV is an infectious disease caused by the hepatitis C virus, which primarily affects the liver, causing liver cirrhosis and carcinoma. The virus that causes HCV is mainly transmitted to humans by the transfusion of human body fluids, similarly to HIV [148]. In the past, it was shown that people with HCV who used Cannabis had a higher risk of advanced liver fibrosis [149,150,151]. However, it has recently been shown that Cannabis use has no impact on the development of liver fibrosis in patients with HCV infection. Rather, it has been shown that daily Cannabis use can protect against the development of fatty liver disease in HIV-infected patients [152].

*COVID-19* (*Coronavirus Disease 19)* is caused by a new virus, the severe acute respiratory syndrome coronavirus 2 (SARS-CoV-2) [153,154,155]. SARS-CoV-2 infection occurs by aerosol transmission/droplets through direct contact with an infected person. The virus enters the body through the epithelial cells of the tongue, bronchi, and lungs after attaching itself to the angiotensin-converting enzyme 2 (ACE2). An important function of soluble, membrane-bound ACE2 is the degradation of angiotensin II (Ang II) to angiotensin 1-7 (Ang 1-7). The affinity of SARS-CoV-2 with ACE2 is determined by the S1 glycoprotein located on the characteristic viral “peaks”. S1 binds to the enzyme via its receptor-binding domain to the transmembrane of serine protease 2 (TMPRSS2), which allows the virus to enter the cytoplasm of host cells [152,153,156,157].

The use of corticosteroids and hydroxychloroquine for the treatment of COVID-19 is not universally successful. Therefore, it is important to seek additional treatment options. One such option could be cannabinoids, and CBD could be a candidate, mainly due to its anti-inflammatory and antioxidant properties [158,159]. These properties are responsible for its high therapeutic potential in the prevention and treatment of a cytokine storm, pneumonia, and ARDS. In models of diabetic cardiomyopathy [160], autoimmune myocarditis [161], and asthma [162], CBD has shown antifibrotic properties. For this reason, CBD may be a potential antifibrotic agent in the treatment of COVID-19 convalescents who develop pulmonary fibrosis [163]. However, most of the tests have been performed on animal models; it is necessary to confirm these results in humans. In vitro testing using CBD-rich Cannabis sativa extracts with a small blend of Δ9-THC on human airway epithelium reduced the expression of the transmembrane serine protease 2 (TMPRSS2) and enzyme ACE2 [164]. In in vivo cell culture models, CBD and Δ9-THC showed antiviral activity through the inhibition of SARS-CoV-2 translation. However, it should be mentioned that at high doses, these compounds are cytotoxic to host cells, which, in addition to the psychoactive properties of Δ9-THC, is one of the reasons why their use in medicine is limited [165]. In the treatment of COVID-19, CB2 receptor agonists should be considered potential agents in the treatment of a cytokine storm due to their strong anti-inflammatory and immunosuppressive properties. Currently, no studies have been conducted using CB2 receptor agonists in SARS-CoV-2 infections. However, these compounds alleviated the pathological changes seen in several respiratory disease models, including viral infection models. It should be noted that the anti-inflammatory properties of cannabinoids are not always beneficial in viral diseases. Suppression of the inflammatory reaction in some viral infections can lead to increased replication of the virus, exacerbation of the disease, and even death. Furthermore, damage to the respiratory epithelium increases the risk of concomitant bacterial infections and sepsis in patients with COVID-19 [166,167].

## 6. Conclusions and Future Perspective

Here, we have highlighted clinical evidence supporting the therapeutic use of cannabinoids in medicine for the treatment of diseases. The involvement of the endocannabinoid system in the physiological functions and in the pathological state of diseases has allowed the development of more efficacious and safer cannabinoid-based drugs. Further research is needed to identify promising therapeutic targets within the endocannabinoid system and to investigate the pharmacological effects of minor phytocannabinoids. Clinical results with preparations containing Δ9-THC, CBD, and palmitoylethanolamide are greatly promising for several disorders (neurodegenerative diseases and neuropathic pain, among others). In cancer, the therapeutic utility is still limited to their analgesic and antiemetic properties such as for nausea and vomiting. In conclusion, further exploration and understanding of the potential risks and benefits of cannabinoid-based drugs is needed through large-scale clinical trials to evaluate their therapeutic potential.

Correspondence and requests for materials should be addressed to Chiara Laezza (c.laezza@ieos.cnr.it or chilaez@hotmail.com) or Cristina Pagano pagano.cris@gmail.com or cristina.pagano@unina.it.

## Abbreviations

2-AG	2-arachidonoylglycerol
5HT1A	5-hydroxytryptamin
ACE2	Angiotensin-converting enzyme 2
AD	Alzheimer’s disease
AEA	Anandamide
AIDS	Acquired immunodeficiency syndrome
AJA	Ajulemic acid
Ang 1-7	Angiotensin 1-7
Ang II	Angiotensin II
ART	Antiretroviral therapy
cAMP	Cyclic adenosine monophosphate
CB1	Cannabinoid receptor 1
CB2	Cannabinoid receptor 2
CBC	Cannabichromene
CBD	Cannabidiol
CBG	Cannabigerol
CBM	Cannabinoid-based medicine
CBN	Cannabinol
CNS	Central nervous system
COVID-19	Coronavirus disease 19
CUD	Cannabis use disorder
CYP	Cytochrome P450
DAGL	Diacylglycerol lipase
EAE	Encephalomyelitis
EB	Epidermolysis bullosa
EC	Endocannabinoid
ECS	Endocannabinoid system
ERK	Extracellular signal-regulated kinases
FAAH	Fatty acid amide hydrolase
GPCR55	G-protein-coupled receptor 55
HCV	Hepatitis C virus
HIV	Human immunodeficiency virus
HIV-SN	HIV-associated sensory neuropathy
LGS	Lennox–Gastaut syndrome
MAGL	Monoacylglycerol lipase
MAPK	Mitogen-activated protein kinase
MS	Multiple sclerosis
NAPE-PLD	N-acyl-phosphatidylethanolamine-specific phospholipase D
NAT	N-acyltransferase
NMS	Non-motor symptoms
NNT	Necessary number for treatment
PEA	Palmitoylethanolamide
PI	Protease inhibitor
PI3K	Phosphoinositide 3-kinase
PPARs	Peroxisome proliferator-activated receptors
SARS-CoV-2	Severe acute respiratory syndrome coronavirus 2
SIV	Simian immunodeficiency virus
SNc	Substantia nigra pars compacta
THCV	Tetrahydrocannabivarin
TMPRSS2	Transmembrane of serine protease 2
TRP	Transient receptor potential
TRPA1	Transient receptor potential cation channel, subfamily A, member 1
TRPV1	Transient receptor potential cation channel, subfamily V, member 1
TRPV2	Transient receptor potential cation channel, subfamily V, member 2
UGP	UDP glucuronosyltransferase
UGT	5′-diphosphoglucuronosyltransferase
Δ9-THC or THC-Δ9 Delta-9-tetrahydrocannabinol	

## References

[1] Alves P., Amaral C., Teixeira N., Correia-da-Silva G. (2020). Cannabis sativa: Much more beyond Δ9-tetrahydrocannabinol. Pharmacol. Res..

[2] Maccarrone M. (2020). Missing Pieces to the Endocannabinoid Puzzle. Trends Mol. Med..

[3] Matsuda L.A., Lolait S.J., Brownstein M.J., Young A.C., Bonner T.I. (1990). Structure of a cannabinoid receptor and functional expression of the cloned cDNA. Nature.

[4] Zou S., Kumar U. (2018). Cannabinoid Receptors and the Endocannabinoid System: Signaling and Function in the Central Nervous System. Int. J. Mol. Sci..

[5] Munro S., Thomas K.L., Abu-Shaar M. (1993). Molecular characterization of a peripheral receptor for cannabinoids. Nature.

[6] Lowe H., Toyang N., Steele B., Bryant J., Ngwa W. (2021). The Endocannabinoid System: A Potential Target for the Treatment of Various Diseases. Int. J. Mol. Sci..

[7] Pertwee R.G. (2005). Pharmacological actions of cannabinoids. Cannabinoids.

[8] Morales P., Hurst D.P., Reggio P.H. (2017). Molecular Targets of the Phytocannabinoids: A Complex Picture. Prog. Chem. Org. Nat. Prod..

[9] Laezza C., Pagano C., Navarra G., Pastorino O., Proto M.C., Fiore D., Piscopo C., Gazzerro P., Bifulco M. (2020). The Endocannabinoid System: A Target for Cancer Treatment. Int. J. Mol. Sci..

[10] Pagano C., Navarra G., Coppola L., Bifulco M., Laezza C. (2021). Molecular Mechanism of Cannabinoids in Cancer Progression. Int. J. Mol. Sci..

[11] Jones É., Vlachou S. (2020). A Critical Review of the Role of the Cannabinoid Compounds Δ^9^-Tetrahydrocannabinol (Δ^9^-THC) and Cannabidiol (CBD) and their Combination in Multiple Sclerosis Treatment. Molecules.

[12] Nahas G.G., Frick H.C., Lattimer J.K., Latour C., Harvey D. (2002). Pharmacokinetics of THC in brain and testis, male gametotoxicity and premature apoptosis of spermatozoa. Hum. Psychopharmacol. Clin. Exp..

[13] Huestis M.A. (2007). Human Cannabinoid Pharmacokinetics. Chem. Biodivers..

[14] McGilveray I.J. (2005). Pharmacokinetics of Cannabinoids. Pain Res. Manag..

[15] Pisanti S., Malfitano A.M., Ciaglia E., Lamberti A., Ranieri R., Cuomo G., Abate M., Faggiana G., Proto M.C., Fiore D. (2017). Cannabidiol: State of the art and new challenges for therapeutic applications. Pharmacol. Ther..

[16] Carrier E.J., Auchampach J.A., Hillard C.J. (2006). Inhibition of an equilibrative nucleoside transporter by cannabidiol: A mechanism of cannabinoid immunosuppression. Proc. Natl. Acad. Sci. USA.

[17] Espejo-Porras F., Fernández-Ruiz J., Pertwee R.G., Mechoulam R., García C. (2013). Motor effects of the non-psychotropic phytocannabinoid cannabidiol that are mediated by 5-HT1A receptors. Neuropharmacology.

[18] Bisogno T., Hanuš L., De Petrocellis L., Tchilibon S., Ponde E.D., Brandi I., Moriello A.S., Davis J.B., Mechoulam R., Di Marzo V. (2001). Molecular targets for cannabidiol and its synthetic analogues: Effect on vanilloid VR1 receptors and on the cellular uptake and enzymatic hydrolysis of anandamide. J. Cereb. Blood Flow Metab..

[19] Černe K. (2020). Toxicological properties of Δ9-tetrahydrocannabinol and cannabidiol. Arch. Ind. Hyg. Toxicol..

[20] Huestis M.A., Solimini R., Pichini S., Pacifici R., Carlier J., Busardò F.P. (2019). Cannabidiol Adverse Effects and Toxicity. Curr. Neuropharmacol..

[21] Thapa D., Cairns E.A., Szczesniak A.-M., Toguri J.T., Caldwell M.D., Kelly M.E.M. (2018). The Cannabinoids Δ8THC, CBD, and HU-308 Act via Distinct Receptors to Reduce Corneal Pain and Inflammation. Cannabis Cannabinoid Res..

[22] Weydt P., Hong S., Witting A., Möller T., Stella N., Kliot M. (2005). Cannabinol delays symptom onset in SOD1 (G93A) transgenic mice without affecting survival. Amyotroph. Lateral Scler..

[23] Borrelli F., Fasolino I., Romano B., Capasso R., Maiello F., Coppola D., Orlando P., Battista G., Pagano E., Di Marzo V. (2013). Beneficial effect of the non-psychotropic plant cannabinoid cannabigerol on experimental inflammatory bowel disease. Biochem. Pharmacol..

[24] Udoh M., Santiago M., Devenish S., McGregor I.S., Connor M. (2019). Cannabichromene is a cannabinoid CB 2 receptor agonist. J. Cereb. Blood Flow Metab..

[25] Cascio M.G., Zamberletti E., Marini P., Parolaro D., Pertwee R.G. (2015). The phytocannabinoid, Δ9-tetrahydrocannabivarin, can act through 5-HT1Areceptors to produce antipsychotic effects. J. Cereb. Blood Flow Metab..

[26] Zamberletti E., Rubino T., Parolaro D. (2021). Therapeutic potential of cannabidivarin for epilepsy and autism spectrum disorder. Pharmacol. Ther..

[27] Fernández-Ruiz J., Galve-Roperh I., Sagredo O., Guzmán M. (2020). Possible therapeutic applications of cannabis in the neuropsychopharmacology field. Eur. Neuropsychopharmacol..

[28] Manera C., Bertini S. (2021). Cannabinoid-Based Medicines and Multiple Sclerosis. Adv. Exp. Med. Biol..

[29] Sholler D.J., Huestis M.A., Amendolara B., Vandrey R., Cooper Z.D. (2020). Therapeutic potential and safety considerations for the clinical use of synthetic cannabinoids. Pharmacol. Biochem. Behav..

[30] Bourgeois B.F.D., Douglass L.M., Sankar R. (2014). Lennox-Gastaut syndrome: A consensus approach to differential diagnosis. Epilepsia.

[31] Golub V., Reddy D.S. (2021). Cannabidiol Therapy for Refractory Epilepsy and Seizure Disorders. Adv. Exp. Med. Biol..

[32] Dravet C., Bureau M., Oguni H., Fukuyama Y., Cokar O. (2005). Severe myoclonic epilepsy in infancy: Dravet syndrome. Adv. Neurol..

[33] Keating G.M. (2017). Delta-9-Tetrahydrocannabinol/Cannabidiol Oromucosal Spray (Sativex^®^): A Review in Multiple Sclerosis-Related Spasticity. Drugs.

[34] Senga S.S., Grose R.P. (2021). Hallmarks of cancer—the new testament. Open Biol..

[35] Aggarwal S. (2016). Use of Cannabinoids in Cancer Care: Palliative Care. Curr. Oncol..

[36] Mackie K. (2005). Distribution of cannabinoid receptors in the central and peripheral nervous system. Cannabinoids.

[37] Nersesyan H., Slavin K.V. (2007). Current approach to cancer pain management: Availability and implications of different treatment options. Ther. Clin. Risk Manag..

[38] Blake A., Wan B.A., Malek L., De Angelis C., Diaz P., Lao N., Chow E., O’Hearn S. (2017). A selective review of medical cannabis in cancer pain management. Ann. Palliat. Med..

[39] Russo E.B. (2007). History of Cannabis and Its Preparations in Saga, Science, and Sobriquet. Chem. Biodivers..

[40] Vučković S., Srebro D., Vujović K.S., Vučetić C., Prostran M. (2018). Cannabinoids and Pain: New Insights from Old Molecules. Front. Pharmacol..

[41] Langford R.M., Mares J., Novotna A., Vachova M., Novakova I., Notcutt W., Ratcliffe S. (2013). A double-blind, randomized, placebo-controlled, parallel-group study of THC/CBD oromucosal spray in combination with the existing treatment regimen, in the relief of central neuropathic pain in patients with multiple sclerosis. J. Neurol..

[42] Guindon J., Hohmann A.G. (2008). Cannabinoid CB2receptors: A therapeutic target for the treatment of inflammatory and neuropathic pain. J. Cereb. Blood Flow Metab..

[43] De Vries M., Van Rijckevorsel D.C., Wilder-Smith O.H., Van Goor H. (2014). Dronabinol and chronic pain: Importance of mechanistic considerations. Expert Opin. Pharmacother..

[44] Boivin M., Narouze S.N. (2021). Nabiximols (Sativex^®^). Cannabinoids and Pain.

[45] https://www.canada.ca/en/health-canada/services/drugs-health-products/medeffect-canada/health-product-infowatch/january-2020.html#shr-pg.

[46] PDQ Supportive and Palliative Care Editorial Board (2002). Nausea and vomiting related to cancer treatment (PDQ^®^): Health professional version. 2021 Nov 22. PDQ Cancer Information Summaries.

[47] Sharkey K.A., Wiley J.W. (2016). The Role of the Endocannabinoid System in the Brain-Gut Axis. Gastroenterology.

[48] Sharkey K.A., Darmani N.A., Parker L.A. (2014). Regulation of nausea and vomiting by cannabinoids and the endocannabinoid system. Eur. J. Pharmacol..

[49] Limebeer C.L., Rock E.M., Mechoulam R., Parker L.A. (2012). The anti-nausea effects of CB1 agonists are mediated by an action at the visceral insular cortex. Br. J. Pharmacol..

[50] (2012). LiverTox: Clinical and Research Information on Drug-Induced Liver Injury. Bethesda (MD): National Institute of Diabetes and Digestive and Kidney Diseases. https://www.ncbi.nlm.nih.gov/books/NBK547865/.

[51] Gaisey J., Narouze S.N., Narouze S.N. (2021). Dronabinol (Marinol^®^). Cannabinoids and Pain.

[52] https://www.canada.ca/en/health-canada/services/drugs-health-products/medical-use-marijuana/information-medical-practitioners/information-health-care-professionals-cannabis-marihuana-marijuana-cannabinoids.html#chp10.

[53] Pisanti S., Picardi P., D’Alessandro A., Laezza C., Bifulco M. (2013). The endocannabinoid signaling system in cancer. Trends Pharmacol. Sci..

[54] Ladin D.A., Soliman E., Griffin L., Van Dross R. (2016). Preclinical and Clinical Assessment of Cannabinoids as Anti-Cancer Agents. Front. Pharmacol..

[55] Javid F.A., Phillips R.M., Afshinjavid S., Verde R., Ligresti A. (2016). Cannabinoid pharmacology in cancer research: A new hope for cancer patients?. Eur. J. Pharmacol..

[56] Salazar M., Carracedo A., Salanueva J., Hernández-Tiedra S., Lorente M., Egia A., Vázquez P., Blázquez C., Torres S., García S. (2009). Cannabinoid action induces autophagy-mediated cell death through stimulation of ER stress in human glioma cells. J. Clin. Investig..

[57] Casanova M.L., Blázquez C., Martínez-Palacio J., Villanueva C., Fernández-Aceñerp M.J., Huffman J.W., Jorcano J.L., Guzmán M. (2003). Inhibition of skin tumor growth and angiogenesis in vivo by activation of cannabinoid receptors. J. Clin. Investig..

[58] Solinas M., Massi P., Cantelmo A., Cattaneo M., Cammarota R., Bartolini D., Cinquina V., Valenti M., Vicentini L., Noonan D. (2012). Cannabidiol inhibits angiogenesis by multiple mechanisms. J. Cereb. Blood Flow Metab..

[59] Preet A., Ganju R.K., E Groopman J. (2008). Δ9-Tetrahydrocannabinol inhibits epithelial growth factor-induced lung cancer cell migration in vitro as well as its growth and metastasis in vivo. Oncogene.

[60] Ramer R., Merkord J., Rohde H., Hinz B. (2010). Cannabidiol inhibits cancer cell invasion via upregulation of tissue inhibitor of matrix metalloproteinases-1. Biochem. Pharmacol..

[61] Twelves C., Sabel M., Checketts D., Miller S., Tayo B., Jove M., Brazil L., Short S.C. (2021). A phase 1b randomised, placebo-controlled trial of nabiximols cannabinoid oromucosal spray with temozolomide in patients with recurrent glioblastoma. Br. J. Cancer.

[62] Guzmán M., Duarte M.J., Blázquez C., Ravina J., Rosa M.C., Galve-Roperh I., Sanchez C., Velasco G., González-Feria L. (2006). A pilot clinical study of Δ9-tetrahydrocannabinol in patients with recurrent glioblastoma multiforme. Br. J. Cancer.

[63] Briggs R., Kennelly S.P., O’Neill D. (2016). Drug treatments in Alzheimer’s disease. Clin. Med..

[64] Amin R., Ali D.W. (2019). Pharmacology of Medical Cannabis. Recent Advances in Cannabinoid Physiology and Pathology.

[65] Russo E.B. (2018). Cannabis Therapeutics and the Future of Neurology. Front. Integr. Neurosci..

[66] Ramírez B.G., Blázquez C., Del Pulgar T.G., Guzmán M., de Ceballos M.L. (2005). Prevention of Alzheimer’s Disease Pathology by Cannabinoids: Neuroprotection Mediated by Blockade of Microglial Activation. J. Neurosci..

[67] Cristino L., Bisogno T., Di Marzo V. (2020). Cannabinoids and the expanded endocannabinoid system in neurological disorders. Nat. Rev. Neurol..

[68] Benito C., Núñez E., Tolón R.M., Carrier E.J., Rábano A., Hillard C.J., Romero J. (2003). Cannabinoid CB2 receptors and fatty acid amide hydrolase are selectively overexpressed in neuritic plaque-associated glia in Alzheimer’s disease brains. J. Neurosci..

[69] Esposito G., De Filippis D., Carnuccio R., Izzo A., Iuvone T. (2006). The marijuana component cannabidiol inhibits β-amyloid-induced tau protein hyperphosphorylation through Wnt/β-catenin pathway rescue in PC12 cells. Klin. Wochenschr..

[70] Aso E., Sánchez-Pla A., Vegas-Lozano E., Maldonado R., Ferrer I. (2015). Cannabis-Based Medicine Reduces Multiple Pathological Processes in AβPP/PS1 Mice. J. Alzheimer’s Dis..

[71] Passmore M.J. (2008). The cannabinoid receptor agonist nabilone for the treatment of dementia-related agitation. Int. J. Geriatr. Psychiatry.

[72] Cohen L.M., Ash E., Outen J.D., Vandrey R., Amjad H., Agronin M., Burhanullah M.H., Walsh P., Wilkins J.M., Leoutsakos J.M. (2021). Study rationale and baseline data for pilot trial of dronabinol adjunctive treatment of agitation in Alzheimer’s dementia (THC-AD). Int. Psychogeriatr..

[73] Volicer L., Stelly M., Morris J., McLaughlin J., Volicer B.J. (1997). Effects of dronabinol on anorexia and disturbed behavior in patients with Alzheimer’s disease. Int. J. Geriatr. Psychiatry.

[74] Galvan A., Wichmann T. (2008). Pathophysiology of Parkinsonism. Clin. Neurophysiol..

[75] Skaper S.D., Facci L., Zusso M., Giusti P. (2018). An Inflammation-Centric View of Neurological Disease: Beyond the Neuron. Front. Cell. Neurosci..

[76] Bortolanza M., Nascimento G.C., Socias S.B., Ploper D., Chehín R.N., Raisman-Vozari R., Del-Bel E. (2018). Tetracycline repurposing in neurodegeneration: Focus on Parkinson’s disease. J. Neural Transm..

[77] Rojo-Bustamante E., Abellanas M., Clavero P., Thiolat M.-L., Li Q., Luquin M.R., Bezard E., Aymerich M.S. (2018). The expression of cannabinoid type 1 receptor and 2-arachidonoyl glycerol synthesizing/degrading enzymes is altered in basal ganglia during the active phase of levodopa-induced dyskinesia. Neurobiol. Dis..

[78] Hurley M.J., Mash D.C., Jenner P. (2003). Expression of cannabinoid CB 1 receptor mRNA in basal ganglia of normal and parkinsonian human brain. J. Neural Transm..

[79] Pisani A., Fezza F., Galati S., Battista N., Napolitano S., Finazzi-Agrò A., Bernardi G., Brusa L., Pierantozzi M., Stanzione P. (2005). High endogenous cannabinoid levels in the cerebrospinal fluid of untreated Parkinson’s disease patients. Ann. Neurol..

[80] Gubellini P., Picconi B., Bari M., Battista N., Calabresi P., Centonze D., Bernardi G., Finazzi-Agrò A., Maccarrone M. (2002). Experimental Parkinsonism Alters Endocannabinoid Degradation: Implications for Striatal Glutamatergic Transmission. J. Neurosci..

[81] Fraguas-Sánchez A.I., Torres-Suárez A.I. (2018). Medical Use of Cannabinoids. Drugs.

[82] Buhmann C., Mainka T., Ebersbach G., Gandor F. (2019). Evidence for the use of cannabinoids in Parkinson’s disease. J. Neural Transm..

[83] Zuardi A.W., Crippa J.A.S., Hallak J.E.C., Pinto J.P., Chagas M.H.N., Rodrigues G.R., Dursun S.M., Tumas V. (2009). Cannabidiol for the treatment of psychosis in Parkinson’s disease. J. Psychopharmacol..

[84] Peball M., Krismer F., Knaus H., Djamshidian A., Werkmann M., Carbone F., Ellmerer P., Heim B., Marini K., Valent D. (2020). Non-Motor Symptoms in Parkinson’s Disease are Reduced by Nabilone. Ann. Neurol..

[85] Brotini S., Schievano C., Guidi L. (2017). Ultra-Micronized Palmitoylethanolamide: An Efficacious Adjuvant Therapy for Parkinson’s Disease. CNS Neurol. Disord. Drug Targets.

[86] McGinley M.P., Goldschmidt C.H., Rae-Grant A.D. (2021). Diagnosis and Treatment of Multiple Sclerosis. JAMA J. Am. Med Assoc..

[87] Chiurchiù V., van der Stelt M., Centonze D., Maccarrone M. (2018). The endocannabinoid system and its therapeutic exploitation in multiple sclerosis: Clues for other neuroinflammatory diseases. Prog. Neurobiol..

[88] Di Filippo M., Pini A.L., Pelliccioli G.P., Calabresi P., Sarchielli P. (2008). Abnormalities in the cerebrospinal fluid levels of endocannabinoids in multiple sclerosis. J. Neurol. Neurosurg. Psychiatry.

[89] Arevalo-Martin A., Vela J.M., Molina-Holgado E., Borrell J., Guaza C. (2003). Therapeutic Action of Cannabinoids in a Murine Model of Multiple Sclerosis. J. Neurosci..

[90] Elliott D.M., Singh N., Nagarkatti M., Nagarkatti P.S. (2018). Cannabidiol Attenuates Experimental Autoimmune Encephalomyelitis Model of Multiple Sclerosis Through Induction of Myeloid-Derived Suppressor Cells. Front. Immunol..

[91] Orefice N.S., Al Houayek M., Carotenuto A., Montella S., Barbato F., Comelli A., Calignano A., Muccioli G.G., Orefice G. (2016). Oral palmitoylethanolamide treatment is associated with reduced cutaneous adverse effects of interferon-β1a and circulating proinflammatory cytokines in relapsing–remitting multiple sclerosis. Neurotherapeutics.

[92] Baswan S.M., Klosner A.E., Glynn K., Rajgopal A., Malik K., Yim S., Stern N. (2020). Therapeutic Potential of Cannabidiol (CBD) for Skin Health and Disorders. Clin. Cosmet. Investig. Dermatol..

[93] Milando R., Friedman A. (2019). Cannabinoids: Potential Role in Inflammatory and Neoplastic Skin Diseases. Am. J. Clin. Dermatol..

[94] del Río C., Millán E., García V., Appendino G., DeMesa J., Muñoz E. (2018). The endocannabinoid system of the skin. A potential approach for the treatment of skin disorders. Biochem. Pharmacol..

[95] Sivesind T.E., Runion T., Branda M., Schilling L.M., Dellavalle R.P. (2021). Dermatologic Research Potential of the Observational Health Data Sciences and Informatics (OHDSI) Network. Dermatology.

[96] Tóth K.F., Ádám D., Bíró T., Oláh A. (2019). Cannabinoid Signaling in the Skin: Therapeutic Potential of the “C(ut)annabinoid” System. Molecules.

[97] Chelliah M.P., Zinn Z., Khuu P., Teng J.M.C. (2018). Self-initiated use of topical cannabidiol oil for epidermolysis bullosa. Pediatr. Dermatol..

[98] Schräder N., Duipmans J., Molenbuur B., Wolff A., Jonkman M. (2019). Combined tetrahydrocannabinol and cannabidiol to treat pain in epidermolysis bullosa: A report of three cases. Br. J. Dermatol..

[99] Scheau C., Badarau I.A., Mihai L.-G., Scheau A.-E., Costache D.O., Constantin C., Calina D., Caruntu C., Costache R.S., Caruntu A. (2020). Cannabinoids in the Pathophysiology of Skin Inflammation. Molecules.

[100] Ali A., Akhtar N. (2015). The safety and efficacy of 3% Cannabis seeds extract cream for reduction of human cheek skin sebum and erythema content. Pak. J. Pharm. Sci..

[101] Dobrosi N., Tóth B.I., Nagy G., Dózsa A., Géczy T., Nagy L., Zouboulis C.C., Paus R., Kovács L., Bíró T. (2008). Endocannabinoids enhance lipid synthesis and apoptosis of human sebocytes via cannabinoid receptor-2-mediated signaling. FASEB J..

[102] Oláh A., Markovics A., Szabó-Papp J., Szabó P.T., Stott C., Zouboulis C.P.D., Bíró T. (2016). Differential effectiveness of selected non-psychotropic phytocannabinoids on human sebocyte functions implicates their introduction in dry/seborrhoeic skin and acne treatment. Exp. Dermatol..

[103] Evaluation of BTX 1503 in Patients with Moderate to Severe Acne Vulgaris. https://clinicaltrials.gov/ct2/show/NCT03573518.

[104] Cooper E., Callahan M. (2018). Patent No: WO2018148786A1 (Formulations of Cannabinoids for the treatment of Acne) Botanix Pharmaceuticals Ltd. https://patents.google.com/patent/WO2018148786A1/en.

[105] Burstein S.H. (2018). Ajulemic acid: Potential treatment for chronic inflammation. Pharmacol. Res. Perspect..

[106] Spiera R., Hummers L., Chung L., Frech T., Domsic R., Furst D., Gordon J., Mayes M., Simms R., Constantine S. (2017). OP0126A phase 2 study of safety and efficacy of anabasum (JBT-101) in systemic sclerosis. Ann. Rheum. Dis..

[107] Chen K., Zeidi M., Reddy N., White B., Werth V. (2019). FRI0307 Lenabasum, a Cannabinoid Type 2 Receptor Agonist, Reduces CD4 Cell Population and Downregulates Type 1 and 2 Interferon Activities in Lesional Dermatomyositis Skin. Ann. Rheum. Dis..

[108] Werth V., Oddis C.V., Lundberg I.E., Fiorentino D., Cornwall C., Dgetluck N., Constantine S., White B. (2019). SAT0303 Design of Phase 3 Study of Lenabasum for the Treatment of Dermatomyositis. Ann. Rheum. Dis..

[109] Yuan C., Wang X.M., Guichard A., Tan Y.M., Qian C.Y., Yang L.J., Humbert P. (2014). N-palmitoylethanolamine and N-acetylethanolamine are effective in asteatotic eczema: Results of a randomized, double-blind, controlled study in 60 patients. Clin. Interv. Aging.

[110] Maghfour J., Rietcheck H., Rundle C., Runion T., Jafri Z., Dercon S., Lio P., Fernandez J., Fujita M., Dellavalle R. (2020). An Observational Study of the Application of a Topical Cannabinoid Gel on Sensitive Dry Skin. J. Drugs Dermatol..

[111] Pulvirenti N., Nasca M.R., Micali G. (2007). Topical adelmidrol 2% emulsion, a novel aliamide, in the treatment of mild atopic dermatitis in pediatric subjects: A pilot study. Acta Dermatovenerol. Croat..

[112] Maida V., Corban J. (2017). Topical Medical Cannabis: A New Treatment for Wound Pain—Three Cases of Pyoderma Gangrenosum. J. Pain Symptom Manag..

[113] Friedman A., Momeni K., Kogan M. (2020). Topical Cannabinoids for the Management of Psoriasis Vulgaris: Report of a Case and Review of the Literature. J. Drugs Dermatol..

[114] Palmieri B., Laurino C., Vadalà M. (2019). A therapeutic effect of cbd-enriched ointment in inflammatory skin diseases and cutaneous scars. Clin. Ter..

[115] Weiss R.A., Colonna M., Brooks E., Falco M., Ferrara G., Strominger J. (1993). How Does HIV Cause AIDS?. Science.

[116] Kalayjian R.C., Machekano R.N., Rizk N., Robbins G.K., Gandhi R.T., Rodriguez B.A., Pollard R.B., Lederman M.M., Landay A. (2010). Pretreatment levels of soluble cellular receptors and interleukin-6 are associated with HIV disease progression in subjects treated with highly active antiretroviral therapy. J. Infect. Dis..

[117] Funderburg N., Mayne E., Sieg S.F., Asaad R., Jiang W., Kalinowska M., Luciano A.A., Stevens W., Rodriguez B., Brenchley J.M. (2010). Increased tissue factor expression on circulating monocytes in chronic HIV infection: Relationship to in vivo coagulation and immune activation. Blood.

[118] Brenchley J.M., Price D.A., Schacker T.W., Asher T.E., Silvestri G., Rao S., Kazzaz Z., Bornstein E., Lambotte O., Altmann D. (2006). Microbial translocation is a cause of systemic immune activation in chronic HIV infection. Nat. Med..

[119] Estes J.D., Harris L.D., Klatt N.R., Tabb B., Pittaluga S., Paiardini M., Barclay G.R., Smedley J., Pung R., Oliveira K.M. (2010). Damaged Intestinal Epithelial Integrity Linked to Microbial Translocation in Pathogenic Simian Immunodeficiency Virus Infections. PLoS Pathog..

[120] Costiniuk C.T., Angel J.B. (2012). Human immunodeficiency virus and the gastrointestinal immune system: Does highly active antiretroviral therapy restore gut immunity?. Mucosal Immunol..

[121] Chun T.-W., Carruth L., Finzi D., Shen X., DiGiuseppe J.A., Taylor H., Hermankova M., Chadwick K., Margolick J., Quinn T.C. (1997). Quantification of latent tissue reservoirs and total body viral load in HIV-1 infection. Nature.

[122] Chomont N., El-Far M., Ancuta P., Trautmann L., Procopio F.A., Yassine-Diab B., Boucher G., Boulassel M.-R., Ghattas G., Brenchley J.M. (2009). HIV reservoir size and persistence are driven by T cell survival and homeostatic proliferation. Nat. Med..

[123] Chomont N., DaFonseca S., Vandergeeten C., Ancuta P., Sékaly R.-P. (2011). Maintenance of CD4+ T-cell memory and HIV persistence: Keeping memory, keeping HIV. Curr. Opin. HIV AIDS.

[124] Zurier R.B., Burstein S.H. (2016). Cannabinoids, inflammation, and fibrosis. FASEB J..

[125] Abrams D.I., Hilton J.F., Leiser R.J., Shade S.B., Elbeik T.A., Aweeka F.T., Benowitz N.L., Bredt B.M., Kosel B., Aberg J.A. (2003). Short-Term Effects of Cannabinoids in Patients with HIV-1 Infection. Ann. Intern. Med..

[126] Lee M.H., Hancox R.J. (2011). Effects of smoking cannabis on lung function. Expert Rev. Respir. Med..

[127] Tahamtan A., Tavakoli-Yaraki M., Rygiel T.P., Mokhtari-Azad T., Salimi V. (2015). Effects of cannabinoids and their receptors on viral infections. J. Med. Virol..

[128] Smith T.H., Sim-Selley L.J., Selley E.D. (2010). Cannabinoid CB1 receptor-interacting proteins: Novel targets for central nervous system drug discovery?. J. Cereb. Blood Flow Metab..

[129] DiPatrizio N.V. (2016). Endocannabinoids in the Gut. Cannabis Cannabinoid Res..

[130] Whiting P., Wolff R.F., Deshpande S., Di Nisio M., Duffy S., Hernandez A.V., Keurentjes J.C., Lang S., Misso K., Ryder S. (2015). Cannabinoids for Medical Use. JAMA.

[131] Mallat A., Teixeira-Clerc F., Deveaux V., Manin S., Lotersztajn S. (2011). The endocannabinoid system as a key mediator during liver diseases: New insights and therapeutic openings. J. Cereb. Blood Flow Metab..

[132] Teixeira-Clerc F., Belot M.-P., Manin S., Deveaux V., Cadoudal T., Chobert M.-N., Louvet A., Zimmer A., Tordjmann T., Mallat A. (2010). Beneficial paracrine effects of cannabinoid receptor 2 on liver injury and regeneration. Hepatology.

[133] Muñoz J., Ros J., Fern’ández-Varo G., Tugues S., Morales-Ruiz M., Alvarez C.E., Friedman S.L., Arroyo V., Jiménez W. (2008). Regression of Fibrosis after Chronic Stimulation of Cannabinoid CB2 Receptor in Cirrhotic Rats. J. Pharmacol. Exp. Ther..

[134] Tam J., Vemuri V.K., Liu J., Bátkai S., Mukhopadhyay B., Godlewski G., Osei-Hyiaman D., Ohnuma S., Ambudkar S.V., Pickel J. (2010). Peripheral CB1 cannabinoid receptor blockade improves cardiometabolic risk in mouse models of obesity. J. Clin. Investig..

[135] Cluny N.L., Vemuri V.K., Chambers A.P., Limebeer C.L., Bedard H., Wood J.T., Lutz B., Zimmer A., Parker L.A., Makriyannis A. (2010). A novel peripherally restricted cannabinoid receptor antagonist, AM6545, reduces food intake and body weight, but does not cause malaise, in rodents. Br. J. Pharmacol..

[136] Costantino C., Gupta A., Yewdall A.W., Dale B.M., Devi L.A., Chen B.K. (2012). Cannabinoid Receptor 2-Mediated Attenuation of CXCR4-Tropic HIV Infection in Primary CD4+ T Cells. PLoS ONE.

[137] Green S.T., Nathwani D., Goldberg D.J., Kennedy D.H. (1989). Nabilone as effective therapy for intractable nausea and vomiting in AIDS. Br. J. Clin. Pharmacol..

[138] Abrams D.I., Jay C.A., Shade S.B., Vizoso H., Reda H., Press S., Kelly M.E., Rowbotham M.C., Petersen K.L. (2007). Cannabis in painful HIV-associated sensory neuropathy: A randomized placebo-controlled trial. Neurology.

[139] Andreae M.H., Carter G.M., Shaparin N., Suslov K., Ellis R., Ware M.A., Abrams D.I., Prasad H., Wilsey B., Indyk D. (2015). Inhaled Cannabis for Chronic Neuropathic Pain: A Meta-analysis of Individual Patient Data. J. Pain.

[140] Ellis R.J., Toperoff W., Vaida F., Brande G.V.D., Gonzales J., Gouaux B., Bentley H., Atkinson J.H. (2009). Smoked Medicinal Cannabis for Neuropathic Pain in HIV: A Randomized, Crossover Clinical Trial. Neuropsychopharmacology.

[141] Phillips T.J.C., Cherry C.L., Cox S., Marshall S.J., Rice A.S.C. (2010). Pharmacological Treatment of Painful HIV-Associated Sensory Neuropathy: A Systematic Review and Meta-Analysis of Randomised Controlled Trials. PLoS ONE.

[142] Riggs P.K., Vaida F., Rossi S.S., Sorkin L.S., Gouaux B., Grant I., Ellis R.J. (2012). A pilot study of the effects of cannabis on appetite hormones in HIV-infected adult men. Brain Res..

[143] Bloom J.W., Kaltenborn W.T., Paoletti P., Camilli A., Lebowitz M.D. (1987). Respiratory effects of non-tobacco cigarettes. BMJ.

[144] Tashkin D.P., Fligiel S., Wu T.C., Gong H., Barbers R.G., Coulson A.H., Simmons M.S., Beals T.F. (1990). Effects of habitual use of marijuana and/or cocaine on the lung. NIDA Res. Monogr..

[145] Yayan J., Rasche K. (2016). Damaging Effects of Cannabis Use on the Lungs. Advancements in Clinical Research.

[146] Tashkin D.P., Baldwin G.C., Sarafian T., Dubinett S., Roth M.D. (2002). Respiratory and Immunologic Consequences of Marijuana Smoking. J. Clin. Pharmacol..

[147] Sherrill D.L., Krzyzanowski M., Bloom J.W., Lebowitz M.D. (1991). Respiratory Effects of Non-Tobacco Cigarettes: A Longitudinal Study in General Population. Int. J. Epidemiology.

[148] Aceijas C., Rhodes T. (2007). Global estimates of prevalence of HCV infection among injecting drug users. Int. J. Drug Policy.

[149] Hezode C., Roudot-Thoraval F., Nguyen S., Grenard P., Julien B., Zafrani E.-S., Pawlostky J.-M., Dhumeaux D., Lotersztajn S., Mallat A. (2005). Daily cannabis smoking as a risk factor for progression of fibrosis in chronic hepatitis C. Hepatology.

[150] Ishida J.H., Peters M.G., Jin C., Louie K., Tan V., Bacchetti P., Terrault N.A. (2008). Influence of Cannabis Use on Severity of Hepatitis C Disease. Clin. Gastroenterol. Hepatol..

[151] Wijarnpreecha K., Panjawatanan P., Ungprasert P. (2018). Use of cannabis and risk of advanced liver fibrosis in patients with chronic hepatitis C virus infection: A systematic review and meta-analysis. J. Evidence-Based Med..

[152] Nordmann S., Vilotitch A., Roux P., Esterle L., Spire B., Marcellin F., Salmon-Ceron D., Dabis F., Chas J., Rey D. (2018). Daily cannabis and reduced risk of steatosis in human immunodeficiency virus and hepatitis C virus-co-infected patients (ANRS CO13-HEPAVIH). J. Viral Hepat..

[153] Baj J., Karakuła-Juchnowicz H., Teresiński G., Buszewicz G., Ciesielka M., Sitarz E., Forma A., Karakuła K., Flieger W., Portincasa P. (2020). COVID-19: Specific and Non-Specific Clinical Manifestations and Symptoms: The Current State of Knowledge. J. Clin. Med..

[154] Tu Y.-F., Chien C.-S., Yarmishyn A.A., Lin Y.-Y., Luo Y.-H., Lin Y.-T., Lai W.-Y., Yang D.-M., Chou S.-J., Yang Y.-P. (2020). A Review of SARS-CoV-2 and the Ongoing Clinical Trials. Int. J. Mol. Sci..

[155] Ali S.A., Baloch M., Ahmed N., Ali A.A., Iqbal A. (2020). The outbreak of Coronavirus Disease 2019 (COVID-19)—An emerging global health threat. J. Infect. Public Health.

[156] Cannalire R., Stefanelli I., Cerchia C., Beccari A.R., Pelliccia S., Summa V. (2020). SARS-CoV-2 Entry Inhibitors: Small Molecules and Peptides Targeting Virus or Host Cells. Int. J. Mol. Sci..

[157] Ni W., Yang X., Yang D., Bao J., Li R., Xiao Y., Hou C., Wang H., Liu J., Yang D. (2020). Role of angiotensin-converting enzyme 2 (ACE2) in COVID-19. Crit. Care.

[158] Atalay S., Jarocka-Karpowicz I., Skrzydlewska E. (2019). Antioxidative and Anti-Inflammatory Properties of Cannabidiol. Antioxidants.

[159] Malinowska B., Baranowska-Kuczko M., Kicman A., Schlicker E. (2021). Opportunities, Challenges and Pitfalls of Using Cannabidiol as an Adjuvant Drug in COVID-19. Int. J. Mol. Sci..

[160] Rajesh M., Mukhopadhyay P., Bátkai S., Patel V., Saito K., Matsumoto S., Kashiwaya Y., Horváth B., Mukhopadhyay B., Becker L. (2010). Cannabidiol Attenuates Cardiac Dysfunction, Oxidative Stress, Fibrosis, and Inflammatory and Cell Death Signaling Pathways in Diabetic Cardiomyopathy. J. Am. Coll. Cardiol..

[161] Lee W.-S., Erdelyi K., Matyas C., Mukhopadhyay P., Varga Z.V., Liaudet L., Haskó G., Cihakova D., Mechoulam R., Pacher P. (2016). Cannabidiol Limits T Cell-Mediated Chronic Autoimmune Myocarditis: Implications to Autoimmune Disorders and Organ Transplantation. Mol. Med..

[162] Vuolo F., Abreu S.C., Michels M., Xisto D.G., Blanco N.G., Hallak J.E., Zuardi A.W., Crippa J.A., Reis C., Bahl M. (2019). Cannabidiol reduces airway inflammation and fibrosis in experimental allergic asthma. Eur. J. Pharmacol..

[163] Esposito G., Pesce M., Seguella L., Sanseverino W., Lu J., Corpetti C., Sarnelli G. (2020). The potential of cannabidiol in the COVID-19 pandemic. J. Cereb. Blood Flow Metab..

[164] Wang B., Kovalchuk A., Li D., Rodriguez-Juarez R., Ilnytskyy Y., Kovalchuk I., Kovalchuk O. (2020). In search of preventative strategies: Novel high-CBD cannabis sativa extracts modulate ACE2 expression in COVID-19 gateway tissues. Aging.

[165] Raj V., Park J.G., Cho K.-H., Choi P., Kim T., Ham J., Lee J. (2021). Assessment of antiviral potencies of cannabinoids against SARS-CoV-2 using computational and in vitro approaches. Int. J. Biol. Macromol..

[166] Rossi F., Tortora C., Argenziano M., Di Paola A., Punzo F. (2020). Cannabinoid Receptor Type 2: A Possible Target in SARS-CoV-2 (CoV-19) Infection?. Int. J. Mol. Sci..

[167] Hill K.P. (2020). Cannabinoids and the Coronavirus. Cannabis Cannabinoid Res..

